# Teenager with acute psychosis due to non-paraneoplastic anti-N-methyl-d-aspartate receptor encephalitis with a successful recovery: A case report

**DOI:** 10.1016/j.amsu.2022.104790

**Published:** 2022-09-28

**Authors:** Rajan Chamlagain, Sangam Shah, Sangharsha Thapa, Madan Basnet, Bipin kandel, Basanta Sharma Paudel, Roman Dhital, Apil Pokhrel, Pitambar Khanal, Sandip Paudel

**Affiliations:** a Institute of Medicine, Tribhuvan University, Maharajgunj, 44600, Nepal; b Kathmandu University School of Medical Sciences, Dhulikhel, Nepal

**Keywords:** Anti NMDAR encephalitis, Anti NMDA receptor Encephalitis, Autoimmune encephalitis

## Abstract

**Introduction:**

Anti NMDAR encephalitis is a neuropsychiatric syndromic disease caused by an immunological response. Acute behavioral changes, psychosis, and catatonia are common clinical manifestations, are seizures, amnesia, speech difficulties, dyskinesia, and autonomic dysregulation.

**Case presentation:**

We discuss the case of a 14-year-old girl who had psychotic symptoms and tested positive for anti-NMDAR antibodies.

**Discussion:**

Patients present with psychiatric symptoms such as delusions, hallucinations, agitation, changes in speech mania, disorganized thinking, catatonia, insomnia, and often seizures. Anti-NMDAR encephalitis should be suspected in teenage patient with acute psychotic symptoms and seizure episodes. A multidisciplinary treatment strategy is required.

**Conclusion:**

The delayed treatment can lead to complications and delayed recovery complicating the disease process so multidisciplinary approach of treatment is necessary.

## Highlights

• Acute psychotic symptoms with seizure episodes in adolescent patient should have a suspicion of anti-NMDAR encephalitis.
• The delayed treatment can lead to complications and delayed recovery complicating the disease process.
• Multidisciplinary approach of treatment is necessary.

## 1 Introduction

N-methyl-d-aspartate receptors (NMDAR) are ligand-gated cation channels for synaptic transmission and plasticity. NR1 subunits of receptor bind glycine whereas NR2 (A, B, C, or D) subunits bind with glutamate [1]. Higher brain processes such as learning and memory are facilitated by this receptor. The hypo-activation of NMDAR results in psychiatric signs and symptoms [2]. Anti NMDAR encephalitis is an acute immune-mediated neuropsychiatric syndromic disorder [3]. It is a rare disease with a prevalence of 1 in 1.5 million [4]. Clinical presentations are acute behavioral change, psychosis, and catatonia along with seizures, amnesia, speech problems, dyskinesia, and autonomic dysregulation. There are five phases of progression: prodromal, psychotic, unresponsive, hyperkinetic, and gradual recovery phase [5]. Patients frequently recover following intensive treatment, immunotherapy, and a lengthier hospital stay with multidisciplinary care, despite the severity of the condition [6]. This case has been reported as per SCARE 2020 criteria [7].

## 2 Case presentation

A previously healthy 14-yr-old girl presented to our hospital with the changes in her behavior which was noticed by her mother. The patient's mother complained of irrelevant talking, visual hallucinations, uncontrollable laughter, weeping, aggressive gazing, and restlessness, as well as decreased interaction with the family and sleeplessness for twenty days. Self-harming activities such as biting buccal mucosa and scratching her body were also reported by her mother. She had urinary and stool incontinence for 10 days with episodic rigidity. There was a history of fever for three days with the maximum recorded temperature being 103 °F without chills and rigors. There had been no unintentional drug consumption, dark urine, traumatic events, abdominal pain, or dog bites in the past. There was no history of loss of consciousness, headache, vomiting, icterus, or body rashes.

She was admitted to a neuropsychiatric ward with a provisional diagnosis of acute psychotic disorder, and olanzapine, lorazepam, and fluoxetine were prescribed. On examination, she was drowsy with glasgow coma scale (GCS) 10/15 with increased tone in the upper and lower limb and hyperreflexia in the patellar region with downgoing plantar reflex. Her total leukocyte count (TLC) was 11,700 cells/cm^3^, neutrophils 78%, lymphocyte 15%, urea 12.9 mg/dl, creatinine 1.04 mg/dl, sodium 151, c-reactive protein (CRP) ++, and erythrocyte sedimentation rate (ESR) was 35 mm in 1st hour. Lumbar puncture revealed lymphocytic pleocytosis in cerebrospinal fluid (CSF) findings as TLC 15/cm^3^, differential leukocyte count (DLC) with 100% lymphocyte, glucose 4.5 mmol/l, and red blood cells (RBC) 150/cm^3^. βhcg, alpha fetoprotein (AFP), anti-nuclear antibody (ANA), and Rh factor were negative. Polymerase chain reaction for COVID-19, HIV and Herpes Simplex Virus ELISA were negative. Thyroid function test was normal. There were no lesions indicative of teratoma on ultrasound of the abdomen and pelvis. The brain magnetic resonance imaging (MRI) was normal, with no noteworthy abnormalities. computed tomography (CT) of chest and abdomen revealed patchy fibrotic changes in apical segment of right upper lobe, apicoposterior segment of left upper lobe, and posterobasal segment of bilateral lower lobes. During the hospital stay in the pediatric ward, she had an episode of urinary tract infection. Urine culture isolated E. coli >100,000 CFU/ml which was treated with iv amikacin 375 mcg and tab cefixime (200mg BD).

She also developed several episodes of seizures during the hospital stay and was prescribed tab levetiracetam (1500mg BD), sodium valproate (1000mg BD), tab and phenobarbital [180mg once a day (OD)]. The child was transferred to the pediatric intensive care unit (PICU) because the seizures were severe and difficult to manage in the ward. During her PICU stay, electroencephalogram (EEG) was done which revealed diffused encephalopathy with generalized very slow basic activity for age without any epileptic discharge. NMDA-R antibodies (NR1) were discovered in her CSF. The final diagnosis of anti NMDAR encephalitis was made. For this, she was treated with tab prednisolone (50mg OD) and injection rituximab [500mg in 450ml Normal Saline (NS) 2 weekly]. With these medications, the patient exhibited remarkable recovery, with no additional seizure episodes. She was discharged after 10 days of steroid and rituximab treatment, with no complications, and was followed up on for the remaining two rituximab doses.

## 3 Discussion

Prevalence of anti NMDAR encephalitis is higher in women with a ratio of 8:2 compared with males and about 37% of patients are children less than 18 years [4]. Tumors (usually ovarian teratoma), and herpes simplex encephalitis are frequent causes of NMDAR encephalitis. COVID-19, Mycoplasma Pneumoniae, Measles, Mumps, Japanese Encephalitis, Toxoplasma Gondi, and Group A Hemolytic Streptococcus are some of the recognized viral infectious causes [[8], [9], [10], [11], [12]]. Approximately 50% of adults and 70% of pediatric patients who present with anti-NMDA receptor encephalitis have no identifiable tumors [4,6,8]. Ovarian teratoma, other neoplasms, and viral etiology were absent in our patient unlike the typical adult patient with anti-NMDA-R encephalitis. The presence of anti-NMDAR Ab in CSF is the most specific marker for diagnosis, while serum NMDAR antibodies are negative in 15% of patients [4,13]. The characteristic electroencephalographic pattern of extreme delta brush, was not seen in our patient. According to Florence et al. out of 32 children, 87.5% presented with behavioral and personality changes also associated with seizures and frequent sleep dysfunction. Titulaer et al. performed the largest cohort study on 577 patients (median age 21 years) for 2 years; 211 out of them were children which showed 90% of patients initially presented with psychiatric and neurological symptoms [8]. One retrospective study by Bigi et al. showed the median time from presentation to diagnosis is 47 days for anti-NMDA receptor encephalitis [14].

Most of the patients are initially evaluated by psychiatrists alone resulting in delayed diagnosis, and initiation of immunotherapy [6,15]. Patients present with psychiatric symptoms such as delusions, hallucinations, agitation, changes in speech mania, disorganized thinking, catatonia, insomnia, and often seizures. Similarly our patient presented with visual hallucination, restlessness insomnia, self-harm and seizures. The approach for anti-NMDA receptor encephalitis should involve multispecialty such as neurology psychiatry, pediatrics and child neurology for early diagnosis and treatment with earlier recovery. Other differential diagnoses for anti-NMDA receptor encephalitis include viral infections (e.g. HSV-1, COVID-19), another auto-immune encephalitis (e.g. Hashimoto's encephalitis or acute demyelinating encephalomyelitis), primary central nervous system vasculitis [16]. Delayed treatment results in hypoventilation, movement abnormalities, dysautonomia, executive dysfunction, impulsivity, disinhibition, and seizures.

There should be a high index of suspicion for anti-NMDA receptor encephalitis if an adolescent presents with a psychotic feature not explained otherwise, as it is treatable with immunotherapy and excision of underlying tumor if present as treatment at early stages improves the outcome [8]. First-line treatment options are high-dose corticosteroids combined with or without IVIG and plasma exchange which usually show improvement after 4 weeks in some patients second-line treatment with rituximab or cyclophosphamide is required [17]. Recovery is good if treated earlier. About 80% of patients improve with immunotherapy. Relapses with the use of serum markers that guide immune-modulating therapy should be monitored.

## 4 Conclusion

Acute psychotic symptoms with seizure episodes in adolescent patient should have a suspicion of anti-NMDAR encephalitis. The delayed treatment can lead to complications and delayed recovery complicating the disease process so multidisciplinary approach of treatment is necessary.

## Provenance and peer review

Not commissioned, externally peer-reviewed.

## Please state any conflicts of interest

Authors have no conflict of interest to declare.

## Please state any sources of funding for your research

No funding was received for the study.

## Ethical approval

None.

## Consent

Written informed consent was obtained from the patient's mother for publication of this case report and accompanying images. A copy of the written consent is available for review by the Editor-in-Chief of this journal on request.

## Author contribution

SS and RC wrote the original draft reviewed and edited the original manuscript. RC, SS, ST, MB, BK, BSP, RD, AP, PK, and SP reviewed and edited the manuscript and were in charge of the case.

## Registration of research studies

1. Name of the registry: None
2. Unique Identifying number or registration ID: None
3. Hyperlink to your specific registration (must be publicly accessible and will be checked): None

## Guarantor

Sangam Shah.
